# Housekeeping by *Leishmania*

**DOI:** 10.1016/j.pt.2006.08.003

**Published:** 2006-10

**Authors:** Paul A. Bates

**Affiliations:** Liverpool School of Tropical Medicine, Pembroke Place, Liverpool, UK, L3 5QA

## Abstract

The observation that differentiation of parasite life cycle stages involves structural and functional reorganisation is very familiar. A recent paper by Besteiro and colleagues provides an intriguing insight into cellular remodelling during differentiation. They show that it is essential for *Leishmania* to tidy up and put the house in order before it can differentiate to mammal-infective stages.

## Introduction

Anyone who has had even a brief encounter with the world of parasites will have quickly realized that the intricacies of their life cycles are one of the most fascinating aspects of parasitology. The ‘what’ of parasite life cycles is well understood in most cases, and is a mainstay of parasitology teaching. However, the ‘how’ still remains largely mysterious. In a recent paper by Besteiro *et al.* [1], the role of two fundamental cell biological processes – endosomal sorting and autophagy – were examined in the life cycle progression of *L. major*, in particular the differentiation of the mammal-infective stages called metacyclic promastigotes (Box 1).

## Investigating endosomes

The authors began by analysing the expression of *L. major* vacuolar protein sorting (VPS)4, a homologue of yeast VPS4 that is an ATPase catalysing the formation of late endosomes. They showed localization of *L. major* VPS4 consistent with an endosomal location. To investigate the function of VPS4, *L. major* expressing a dominant-negative mutant version (VPS4^E235Q^) were created, in which a crucial residue in the active site was changed, rendering the enzyme non-functional. These mutants prematurely entered the stationary phase in culture, were defective in endosomal sorting, and were also unable to differentiate into metacyclic promastigotes.

## Links to autophagy

It has been known for some time that metacyclogenesis (Box 1) and subsequent transformation into amastigotes (Box 1) are both accompanied by large scale changes in protein expression (i.e. significant turnover of cellular proteins). In such situations, cells will often seek to conserve resources by exploiting the autophagy pathway. Therefore, the authors went on to study the functioning of autophagy in the VPS4^E235Q^ mutants by examining the localisation of a GFP-tagged autophagosomal marker (the ubiquitin-like protein ATG8). The mutants possessed more autophagosomes under normal conditions, but these could not be processed properly, and, as a consequence, the mutants were found to be less able to survive nutrient deprivation than wild-type controls, in which autophagy was shown to peak at the initiation of metacyclogenesis. This is a significant finding considering the *in vivo* development of metacyclic promastigotes. The infective stages are the end product of a series of transformation and differentiation steps that occur in the sand fly, and involve at least four different life cycle stages [2]. By the time the metacyclic promastigotes begin to appear during the ‘sugarmeal phase’ of infection (when sand flies are feeding on plant sugars), there are only relatively limited energy and nutrient resources available, compared with the riches of the earlier ‘bloodmeal phase’. Also, the precursors of the metacyclic promastigotes, leptomonad forms, invest considerable resources in the biosynthesis of promastigote secretory gel (PSG), which is a key factor in promoting transmission [3]. Thus, the ability of metacyclic promastigotes to maximise the remaining available energy and nutrient resources by exploiting autophagy enables them to sit and wait for a longer period until the opportunity of transmission by sand fly bites arises.

## Blocking autophagy

Finally, the authors of the paper went on to test directly the importance of the autophagy pathway by creating knockout mutants defective in ATG4 (a cysteine protease that processes ATG8 and is key to the formation of autophagosomes). As predicted, the ATG4 mutants were defective in processing autophagosomes, were vulnerable to starvation and failed to undergo metacyclogenesis, thus independently confirming the results obtained using the VPS4^E235Q^ expressors. Again, it is interesting to relate these findings to the *in vivo* scenario. As the authors note, autophagy is often triggered by high population density, and this probably causes nutrient depletion itself. Mature transmissible infections in sand flies are characterized by extremely high parasite population densities, much higher than is ever achieved *in vitro*, because the parasites are packed into the PSG plug that occludes the anterior midgut [4]. The plug functions as a metacyclic promastigote factory, thus the conditions that are likely to trigger autophagy and the differentiation of metacyclic promastigotes are co-incident *in vivo*. This paper shows that autophagy is an essential prerequisite for metacyclogenesis, whereby the parasites put their own house in order, by removing unwanted organelles and macromolecular complexes, as part of their pre-adapted phenotype for invasion of the mammalian host.

One interesting area that we do not fully understand is what sets this train of events in motion in the first place – what are the signals that trigger metacyclogenesis? Possible signals are low environmental pH [5], low oxygen tension [6] and nutritional depletion of tetrahydrobiopterin [7]. Interestingly, these are all likely to be associated with high population densities. Therefore, it might be possible to use the markers described by Besteiro *et al.* to examine this important part of the molecular aspect of their life cycle. In conclusion, it is satisfying to note that, once again, the study of the kinetoplastids has not only revealed an interesting molecular aspect to their life cycle biology, but also provided another example of an excellent system for examining the function and interaction of basic cell biology processes in eukaryotes.

## Box 1 Definitions of key terms

Amastigotes – non-motile intracellular forms that live inside the phagolysosomal system of mammalian macrophages – a unique location for a eukaryotic parasite. They are true acidophiles, adapted to the low pH of this intracellular compartment.

Autophagy – this is the process by which cells dispose of unwanted and/or damaged organelles or macromolecular complexes, or degrade them as part of a stress response. Destruction of an organelle can be part of a normal cell differentiation process or can be triggered by conditions such as starvation. The organelle is enclosed in membranes in a structure termed an autophagosome, which might then fuse with endocytic vesicles, and ultimately with lysosomes. The battery of lysosomal enzymes degrades the organelle, releasing the nutrients into the cytoplasm for potential re-use in biosynthesis.

Differentiation – the process by which a cell undergoes a change in function, typically by changes in expression of particular genes. In most eukaryotes, differentiation is regulated at the transcriptional level through DNA-binding transcription factors that regulate RNA polymerase–promoter interactions. The situation in kinetoplastids is slightly different but the principle is the same – expression is regulated largely post-transcriptionally by mechanisms that are not yet fully understood. The terms differentiation and transformation are often used interchangeably by kinetoplastid workers. However, they are sometimes used with more-specific meanings: differentiation to describe a change accompanying a cessation of cell division, and transformation to describe a change accompanying a resumption or continuation of cell division. The life cycles of kinetoplastids are characterised by alternation of dividing and non-dividing life cycle stages.

Endosomal sorting – the endosomal pathway is involved in the internalisation of macromolecules from outside the cell, either through surface receptor-mediated mechanisms or pinocytosis. The resulting endocytic vesicles and their contents need to be sorted and sent to the correct destination; for example, to lysosomes for degradation or recycling of receptors back to the cell surface. The endosomal pathway intersects with vesicle traffic from both the exocytic (secretory) pathway and the delivery of lysosomal enzymes following their biosynthesis. All these processes interact with each other directly or indirectly in some way, and must be carefully controlled. In kinetoplastids, like *Leishmania* and *Trypanosoma*, the flagellar pocket plays a particular role, being the main site of exocytosis/endocytosis across the plasma membrane.

Metacyclogenesis – the differentiation of metacyclic promastigotes, which are non-dividing stages. Metacyclic promastigotes are pre-adapted for survival in the mammalian host − they become complement resistant, express stage-specific genes and are biochemically part-way to becoming amastigotes.

Promastigotes – motile extracellular forms that live in the lumen of the sand fly gut and swim by beating their flagella. Various kinds of promastigote occur in the sand fly phase of the life cycle, including metacyclic promastigotes, the mammal-infective stages, and leptomonad promastigotes, their developmental precursors responsible for the synthesis of promastigote secretory gel (PSG).
